# Protección de datos de salud: el reto de la armonización legislativa en América Latina

**Published:** 2023-09-19

**Authors:** Agueda-Muñoz del-Carpio-Toia, Liliana Mondragón-Barrios, Eduardo Alfredo Duro, Laura Rueda Castro, Patricia Sorokin

**Affiliations:** 1 Universidad Católica de Santa María, Vicerrectorado de Investigación, Escuela de Postgrado, Escuela de Medicina Humana, Arequipa, Perú.; 2 Instituto de Ética Clínica Francisco Vallés, Universidad Europea, Madrid, España.; 3 Dirección de Investigaciones Epidemiológicas y Psicosociales, Instituto Nacional de Psiquiatría Ramón de la Fuente, México.; 4 Universidad de Moron, Argentina.; 5 Universidad de Chile. Facultad de Medicina. Departamento Terapia Ocupacional y Ciencia de la Ocupación, Chile; 6 Universidad de Buenos Aires, Argentina; 7 Universidad Abierta Interamericana, Argentina; a Médico Cirujano, Doctora en medicina. Especialización en ética de la investigación; b Master en bioética clínica; c Psicóloga, Doctora en Psicología; d Medico. Especialista en Pediatría, Neonatología y Educación.; e Terapeuta Ocupacional; f Doctora en Ciencias Sociales, Posdoctoral en Protección de Datos.

**Keywords:** Privacidad, datos personales, datos sensibles, salud, pandemia, bioética, Privacy, personal data, sensitive data, health, pandemic, bioethics

## Abstract

La protección de datos personales en los sistemas de salud requiere de medidas y procedimientos especiales para asegurar que la privacidad de la información no sea vulnerada. Los avances en la tecnología digital y el acceso a la transmisión en tiempo real de datos personales, familiares, clínicos y de laboratorio de los pacientes y/o sujetos de estudio, puede comprometer la protección de esta información. La privacidad de los datos personales en salud en tiempos de pandemia ha significado un reto mayor, es por ello que se presenta esta contribución especial que tiene por objetivo, identificar los resguardos éticos y normativos en materia de protección de datos, para garantizar el pleno respeto de los derechos de privacidad de las personas y la confidencialidad de sus datos, bajo el contexto de la atención en salud, sobre todo en condiciones de crisis sanitaria; como la vivida durante la pandemia de SARS-CoV-2. Se propone además una armonización legislativa en América Latina, sobre la privacidad y la protección de datos personales.

## SUMMARY

The protection of personal data in health systems requires special measures and procedures to ensure that the privacy of the information is not violated. Advances in digital technology and access to real-time transmission of personal, family, clinical, and laboratory data of patients and/or study subjects may compromise the protection of this information. The privacy of personal data in health in times of pandemic has meant a greater challenge, that is why this unique contribution is presented, whose objective is to identify the ethical and regulatory safeguards in terms of data protection to guarantee full respect for the privacy rights of people and the confidentiality of their data, under the context of health care, especially in conditions of health crisis, such as the one experienced during the SARS-CoV-2 pandemic. It also proposes a legislative harmonization in Latin America, on privacy and protection of personal data.

## INTRODUCCIÓN

La protección de datos personales en los sistemas de salud implica el despliegue de medidas y procedimientos para asegurar el derecho a la privacidad de la información médica de los pacientes, siendo un derecho personalísimo que se debe resguardar y requiere de la gestión pública, estatal y privada indispensable para lograr una verdadera confidencialidad de estos datos ^(1)^.

A nivel global, la Unión Europea ha dado grandes pasos en la armonización de la legislación de la protección de datos personales. A través del Reglamento General de Protección de Datos (RGPD) del 2018, considera los datos personales como “*cualquier información relativa a una persona física identificada o identificable*” ^(2)^.

El RGPD contempla varios tipos de tratamiento de datos personales, que incluyen los generados en la atención médica y en la investigación biomédica; mediante la posibilidad de la seudoanonimización (en la que el procesamiento de los datos personales se codifica y no pueden atribuirse a una persona, sin el uso de información adicional) y de la anonimización, (cuyo procesamiento de datos personales no permite identificar a la persona) ^(2)^.

Un caso precedente en la región de América Latina ha sido la Ley argentina N° 25.326, actualmente en proceso de actualización, que define a los datos personales como toda información referida a personas físicas y de existencia ideal, determinadas o determinables y considera datos sensibles a aquellos que se refieren tanto a origen racial y étnico, opiniones políticas, convicciones religiosas, filosóficas o morales, afiliación sindical como a la información referente a la salud o a la vida sexual estableciendo a su respecto mayores medidas de protección ^(3)^.

La salvaguarda de los datos personales en salud se establecen de manera precisa en el RGPD, el cual dispone que los servicios de salud aseguren que todo el personal de salud atienda a los pacientes con el debido cuidado de su intimidad, privacidad y respeto de su dignidad, protegiendo los datos médicos. Esta gestión de la confidencialidad de datos personales es parte de las obligaciones legales, deontológicas y éticas que norman el actuar de los profesionales de la salud ^(4)^.

Por otro lado, la repentina aparición de la pandemia por COVID-19 y la necesidad de contenerla, introdujo diversas herramientas digitales para apoyar a las autoridades en la gestión de la crisis sanitaria, tales como la identificación de personas infectadas por SARS-Cov-2 y sus contactos, que constituyeron un riesgo para proteger la confidencialidad de la información personal, pero que en el balance riegos- beneficio, realizado por los gobiernos, desde un enfoque abiertamente utilitarista se inclinaba la balanza a la protección colectiva de la salud versus los derechos individuales. En algunos contextos se llevaron a cabo estrategias aún más rigurosas como la entrega de pasaportes inmunológicos para transitar o trabajar ^(5)^, que causaron dilemas jurídicos y controversias éticas sobre derechos personalísimos, consagrados en diversas declaraciones sobre derechos humanos y bioética, como la privacidad y la confidencialidad cuando aquellas acciones se sobrepusieron a los derechos innatos de las personas, cuya constricción inhibe el desarrollo de sus vidas.

En el contexto de la pandemia, la apremiante necesidad de toma de decisiones basadas en evidencia, llevaron a la transformación digital de los servicios de salud, al manejo diario de gran cantidad de datos (Big Data) y estadísticas sobre las cifras de contagios, hospitalizaciones y fallecidos, que expusieron con mucho riesgo la privacidad de las personas ^(6)^. s transformaciones tecnológicas que se producen de manera exponencial, es necesario contar con instrumentos adecuados para la planificación estratégica, regulación y gestión pública de los Estados Nacionales a fin velar por la protección de los datos personales con el objetivo de construir una mejor sociedad” ^(7)^, se torna imprescindible “armonizar con los estándares regionales e internacionales en materia de protección de datos personales para fortalecer una estrategia global de regulación, desde un enfoque de derechos humanos y con una mirada situada” ^(7)^ con el objeto de garantizar el derecho real y efectivo a la privacidad y a la protección de datos personales y sensibles.

Los “datos a gran escala” y “macrodatos”, Big Data (su significado ya se encuentra globalizado), son la base del creciente y lucrativo comercio digital, debido a que el Big Data puede aplicarse a toda aquella información que no es susceptible de ser procesada y/o analizada mediante el uso de herramientas o procesos tradicionales. Este es el punto que motiva principalmente la reflexión.

El propósito de este trabajo es identificar los resguardos éticos y normativos en materia de protección de datos personales, para garantizar el pleno respeto de los derechos de privacidad de las personas y la confidencialidad de sus datos, bajo el contexto de la atención en salud, sobre todo en condiciones de crisis sanitaria; se propone además una armonización legislativa en América Latina, que permita el fortalecimiento de una estrategia regional de regulación que garantice el derecho a la privacidad y la protección de datos personales, particularmente en el contexto de una crisis sanitaria; como la pandemia por COVID-19.

Como parte del método para obtener la información, se llevó a cabo una revisión de la literatura especializada, no sistemática, para identificar los temas éticos y legislativos en materia de protección de datos personales en América Latina, a través de consultas en internet usando diferentes motores de búsqueda y bases de datos como EBSCO, Web of Science, Elsevier y Google Scholar, PubMed de la U S National Library of Medicine y LILACS de la Organización Panamericana de la Salud, incluyendo acervos documentales legislativos en portales gubernamentales de algunos países como: Argentina, Chile, Colombia, México y Perú, así como de la Unión Europea. Se revisó la información en textos publicados, los cuales fueron analizados sistemática y críticamente e interpretados desde la argumentación bioética, encontrando tres temas relevantes: Riesgos a la privacidad en la atención médica, Privacidad y protección de datos de salud y algunos retos pendientes

### Riesgos a la privacidad en la atención médica

A lo largo de la historia se han evidenciado daños múltiples, en algunos casos irreversibles, a causa de la vulneración de la confidencialidad de los datos de salud, con consecuencias graves como la estigmatización, la discriminación social y el compromiso a la calidad de vida ^(8, 9,)^.

Más aún: muchos datos personalísimos expuestos en las historias clínicas (en papel y digitalizadas) para proveedores y financiadores de servicios, pueden hoy ser nominalizados e incorporados a protocolos de vigilancia epidemiológica. Adicionalmente, pueden estar automatizados y ser recogidos por cualquier persona, generando también riesgos más que mínimos, asociados a la pérdida de confidencialidad.

Debemos considerar que el riesgo de un manejo inadecuado de datos se incrementa en la era del Big Data. Gil González señala que “una persona es identificable cuando, aunque no haya sido identificada todavía, sea posible hacerlo” ^(10)^. En tal sentido, el acceso a la ficha o al expediente clínico electrónico con fines de investigación científica plantea otro posible riesgo a la privacidad en la atención médica, que se encuentra regulado por instrumentos internacionales reconocidos desde años anteriores. Si bien, para la realización de investigaciones de tipo retrospectivo este acceso debe estar restringido a ciertos datos, extraídos de manera anonimizada o asegurando la confidencialidad de estos y con previa autorización legal, en ocasiones los investigadores no especifican en el protocolo, las medidas de protección de los datos sensibles, por ejemplo cuando los estudios abordan enfermedades mentales, algunas infectocontagiosas se agrega al riesgo el tratamiento ilegal de la información, con consecuencias dañinas para las personas, como son la discriminación, la segregación y los perjuicios eventuales de carácter social, educativo, laboral económico, entre otros ^(11,12)^.

La pandemia por COVID-19, no solo trajo muerte, dolor, colapso de los sistemas de salud y serio costo social, liberalizó también la forma de toma de obtención, almacenamiento, transmisión y análisis de datos^(5)^. Pudimos contemplar el uso y abuso de tecnologías de información y comunicación (TIC), de redes sociales y diversas aplicaciones (apps) para notificar en tiempo real, síntomas, resultados de pruebas rápidas, moleculares, estado de vacunación, dispositivos de reconocimiento facial, de termografía para identificar fiebre, entre otros, con el objetivo de contribuir al rastreo de contagios, de contactos y al conocimiento de la situación epidemiológica y catastral individual, familiar ^(13)^, institucional, regional, nacional y global.

En Colombia, al igual que otras naciones de la Región, bajo el contexto de la pandemia, se presentaron alternativas de soporte digital para la vigilancia poblacional; algunas de ellas que abrieron tensiones entre los derechos individuales a la privacidad y los derechos colectivos ^(13).^ Es el caso del uso de la herramienta digital denominada “*Coronapp”:* El gobierno obtenía en tiempo real información auto reportada de manera voluntaria, sobre el estado de salud individual y de la familia en cuanto a síntomas y signos de alarma relacionados con el COVID-19 para monitoreo y georreferencia ^(13)^; situación que deja de garantizar el derecho a la privacidad de los datos personales y de salud, que podrían en ese tiempo haber colaborado a la protección de la salud colectiva de la población pero exponiendo información personalísima obtenida sin resguardos, aunque fuera entregada voluntariamente por personas asustadas y no sin razón.

En México, previo a la pandemia por COVID- 19, se ha sistematizado la protección de los datos personales en el sector público de la salud con una serie de normativas. Durante la emergencia sanitaria, el Instituto Nacional de Transparencia, Acceso a la Información y Protección de Datos Personales (INAI), emitió las recomendaciones Datos Personales Seguros COVID-19, donde se informó sobre el derecho a la protección de datos personales que en la mitigación de la pandemia serían tratados durante el diagnóstico, la atención y el seguimiento del SARS-CoV-2 y su posible contagio, de este modo instó a las instituciones públicas o privadas a llevar a cabo un adecuado tratamiento de datos personales en la atención de casos de COVID-19, cumpliendo la normativa en la materia ^(11,14)^.

No obstante, por el confinamiento por la pandemia el INAI también aplazó de forma indefinida asuntos como impugnación, procedimientos de verificación, imposición de sanciones y denuncias por incumplimiento a las obligaciones de transparencia, provocando la vulneración de los datos personales en salud recolectados en nombre de la pandemia por COVID-19 ^(15)^.

Bajo estas situaciones vulnerables, en México se pusieron en marcha algunos dispositivos parecidos a los de otros países, como el monitoreo y geolocalización de la población a través de una app denominada “*Covidradar*”, desarrollada por una empresa privada que, por ende, manejó datos sensibles de particulares. La relevancia de la emergencia epidemiológica justificó la implementación de estas medidas extraordinarias y con éstas, la eliminación de las garantías de seguridad jurídica relativa al derecho a la privacidad, generando consecuencias graves debido al manejo inadecuado de los datos de salud, cuya vulnerabilidad aún sigue latente para datos personales contenidos en bases informáticas, como el caso de las vacunas contra COVID-19 ^(16)^.

El Ministerio de Salud en Chile (MINSAL) conforme a la Ley 19.628 sobre protección de la vida privada y sus modificaciones posteriores en cuanto al tratamiento de datos personales, determina: asegurar la confidencialidad de los datos personales de los usuarios, entregados voluntariamente, mediante formularios establecidos para esos efectos. A la vez, establece que solo serán conocidos según este indicado en el formulario y las sanciones en caso de su uso con otros fines. Esto rige para toda información digitalizada^(17)^.

Las TIC, aceleradas aún más en el tráfico ocasionado durante la pandemia, requieren en mayor medida la protección de datos personales. Se trata entonces que las personas puedan conocer, editar, gestionar o eventualmente eliminar sus datos; considerando que la protección de éstos implica un sistema de reglas y principios de uso al cual deben sujetarse las empresas e instituciones públicas y privadas.

Perú cuenta con la Ley N° 29733, esta norma reconoce y protege el derecho a la privacidad de los datos personales y la obligación de la toma del consentimiento informado del titular previo a su divulgación; excepto, si se presentan situaciones de riesgo a la salud individual o a la salud pública ^(18)^.

En tiempos de pandemia, en Perú se publicó la Directiva Administrativa 294-MINSA/2020/OGTI, estableciendo protecciones específicas a la privacidad y seguridad de la data personal generada en pandemia, que incluye la confidencialidad de los datos bajo responsabilidad, reubicación de los datos transitorios a historias clínicas correspondientes (si es factible); prohibiendo la eliminación de la información de los pacientes en situaciones de emergencia ^(19)^.

Estas protecciones podrían ser, en algunos casos, de difícil cumplimiento, como la reconstrucción de la historia clínica, debido a las condiciones precarias en las cuales algunos hospitales almacenaron gran cantidad de data en tiempos de pandemia, causadas por las brechas digitales, historias clínicas impresas, problemas de bioseguridad, etc. ^(19)^.

La geolocalización que se implementó para el monitoreo, rastreo o control de la población con datos personales en salud en diversos países de América Latina, utilizó la infraestructura en comunicaciones tanto de los Estados como de empresas privadas principalmente lo referente a la telefonía celular y proveedores de Internet, por ejemplo, el gobierno de Ecuador dispuso de las comunicaciones satelitales para vigilar a las personas que debían estar en aislamiento. Lo problemático de esto es que las TIC garantizan la privacidad de la información, pero el alcance de esta privacidad es limitado, debido a que no considera los imponderables como los hackeos o virus que amenazan y vulneran estos medios ^(16)^.

### Privacidad y protección de datos de salud

La Unión Europea decidió unificar los esfuerzos para armonizar directivas y leyes sobre la privacidad de datos personales, con la finalidad de contar con una efectiva salvaguarda de esta información mediante el RGPD, vigente desde el 2018 ^(2)^. Este reglamento promueve una cultura de protección de datos, regulando las formas cómo las organizaciones deben proteger la privacidad de datos de todos los ciudadanos de la unión europea y se basa en siete principios: “(a) el procesamiento debe darse de manera lícita, justa y transparente; (b) los datos deben ser recopilados para fines específicos, explícitos y legítimos y no procesados de manera incompatible con esos fines; (c) minimización de datos; (d) exactitud; (e) necesaria la limitación de almacenamiento; (f) integridad y confidencialidad; (g) responsabilidad” ^(2)^.

El RGPD propugna que las organizaciones limiten el procesamiento de datos personales y adopten medidas para eliminar la información identificable sensible, siempre que sea posible; sin embargo, bajo el contexto de la investigación, permite el tratamiento secundario de datos personales con fines del estudio, “el cual debe ser compatible con el propósito original para considerarse como lícito” ^(2)^.

En resumen, el RGPD se sustenta bajo dos pilares en cuanto a la necesidad de 1) “procesar datos personales diseñados para servir a la humanidad” y 2) “uso de información de salud personal para la investigación para promover la salud humana” ^(19)^, elementos rectores que garantizan el uso adecuado de estos datos.

El Convenio 108 firmado en el año 1981 por el Consejo de Europa, es considerado como el único instrumento multilateral de carácter vinculante para la protección del tratamiento de datos personales y ha sido actualizado en el año 2018, con la finalidad de enfrentar los nuevos desafíos relacionados a la protección de la privacidad en contextos de mayor acceso a bigdata, a Tics, entre otros y prevenir abusos en el tratamiento de datos personales (Convenio 108+) ^(20)^.

La relevancia de este instrumento internacional radica en ser el único estándar vinculante en materia de protección de datos que tiene el potencial de ser aplicado en todo el mundo, proporcionando seguridad jurídica y previsibilidad en las relaciones internacionales ^(20)^.

### Retos pendientes

Dentro de las medidas de urgencia adoptadas por los gobiernos, prevalecieron los criterios de inmediatez y urgencia, para el manejo de datos en tiempo real de pacientes contagiados por SARS-CoV-2, gracias al avance de las TIC, pero este nuevo escenario, trae consigo diversos riesgos como “la apropiación del dato, y exposición a violaciones de derechos fundamentales” ^(6)^.

Al respecto de este riesgo de apropiación, es necesario recordar en la atención médica en pandemia se generó gran cantidad de datos de pacientes con COVID-19, a través de historias clínicas en hospitalización, unidades de cuidados intensivos, seguimiento domiciliario de pacientes, teleconsultas, campañas de vacunación, innumerables pruebas rápidas y moleculares, geolocalización de contactos, etc.; siendo los riesgos permanentes de esa penetración a la Big Data, los daños a derechos humanos fundamentales, a la dignidad humana, a la privacidad e intimidad, o la apropiación privada del conocimiento ^(6)^.

Un reto aún pendiente es la necesidad de un trabajo colectivo entre los países que conforman América Latina para lograr la armonización de leyes y recomendaciones éticas para la Región (tal como lo ha conseguido la Unión Europea), que aseguren la protección de los datos personales y que respondan a los cambios obligados ocurridos como consecuencia de la crisis sanitaria en la relación médico paciente, en la generación y el acceso de datos, en el incremento de uso de tecnología diversa de TIC, en la implementación de la telemedicina, así como a la fragilidad en los sistemas de salud, expuestos en esta pandemia.

Armonizar una legislación para la región, además de robustecer la garantía a la protección de datos personales, requiere del seguimiento de su cumplimiento y en todo caso, considerar la posibilidad de sancionar el mal manejo de estos datos en posesión de terceros. Por ejemplo, México cuenta con leyes y normas que exigen el ejercicio de los derechos en el acceso y protección de la información de las personas en el ámbito de la salud; no obstante, las sanciones económicas generalmente se aplican en los sectores financiero y del comercio, relacionados con datos no sensibles, mientras que en el campo sanitario no han sido eficientes ^(14–15)^. Como se mencionó anteriormente, el marco normativo existe, pero no se aplica o se hace selectivamente, es decir, eximiendo de consecuencias legales a personas pertenecientes a ciertos sectores que infringen la ley.

No se trata de crear más normativas en cuanto a la protección de datos personales, sino de armonizar la regulación con enfoque global en América Latina que pueda aplicarse rigurosamente y no de forma selectiva, sancionando su incumplimiento en el ámbito de la salud, donde se suelen transgredir los derechos de las personas a la protección sobre su información y datos, principalmente, durante contingencias sanitarias.

Los daños suscitados por la indebida protección de datos sensibles de las personas en situaciones de emergencias sanitarias son irreparables, y en estos casos una sanción no es lo suficientemente compensatoria para subsanar una doble victimización. Por ello, además “se requiere el esfuerzo de organismos internacionales como la ONU [Organización de Naciones Unidas] y la OCDE [Organización para la Cooperación y el Desarrollo Económicos ], que aporten investigación y recursos para contener tecnológicamente las fugas masivas de información” ^(12–16, 21)^.

Finalmente, el desarrollo de medidas específicas relacionadas con la protección de datos personales en los sistemas de salud no debe quedar únicamente en manos de los órganos legislativos nacionales y supranacionales bajo el control y la supervisión de organizaciones de la sociedad civil. En ese sentido, el panorama es sobrio ya que la tecnología de la información avanza más rápido que la reflexión ciudadana y su correlato normativo regulatorio, por lo cual, se debe apelar a la importancia de una incesante cultura de protección de datos por los profesionales de la salud en la atención e investigación para que las utilicen con integridad, responsabilidad, confidencialidad y con el estricto cumplimiento y aplicación de la normativa para todos los casos.

## CONCLUSIONES

Para lograr la armonización de leyes y recomendaciones éticas indispensable para la Región de América Latina se requiere de un trabajo multiparticipativo y representativo, que identifique los principios necesarios para garantizar el tratamiento y la transferencia adecuada de los datos personales y los mecanismos de vigilancia, notificación y sanciones ante vulneraciones a la protección de datos personales y datos sensibles, tanto en contextos de normalidad, como en pandemia; prestando la debida atención a cuestiones como “la mercantilización de los datos personales [que] ha aumentado hasta tal punto que a menudo las personas no son conscientes de la venta o las múltiples reventas de sus datos, ni las consienten completamente, especialmente de los que se consideran confidenciales, como los datos médicos y de salud” ^(22)^.

Esta armonización requiere también de mecanismos transparentes y vinculantes, que brinden seguridad para la transferencia segura y ética de datos entre países que evite la explotación, la violación y los abusos en el uso de la información de las personas, principalmente, cuando se trata de su salud.

Un punto clave, para la vigencia de la ética en el uso de las TIC es que en su diseño se contemplen los valores rectores para proteger a las personas en lo relacionado con la defensa de los derechos fundamentales.
